# Nurses' knowledge of universal health coverage for inclusive and sustainable elderly care services

**DOI:** 10.1590/1518-8345.1152.2670

**Published:** 2016-03-04

**Authors:** Fabian Ling Ngai Tung, Vincent Chun Man Yan, Winnie Ling Yin Tai, Jing Han Chen, Joanne Wai-yee Chung, Thomas Kwok Shing Wong

**Affiliations:** 1 Doctoral student, The Hong Kong Institute of Education, Hong Kong. Researcher, The Hong Kong Institute of Education, Hong Kong; 2 Doctoral student, The City University of Hong Kong, Hong Kong. Researcher, The Hong Kong Institute of Education, Hong Kong; 3 PhD, Researcher, Ginger Knowledge Transfer and Consultancy Limited, Hong Kong; 4 PhD, Full Professor, The Hong Kong Institute of Education, Hong Kong; 5 PhD, Full Professor, Guangzhou University of Chinese Medicine, China

**Keywords:** Universal Coverage, Millennium Development Goals, Health Policy

## Abstract

**Objectives:**

to explore nurses' knowledge of universal health coverage (UHC) for inclusive and sustainable development of elderly care services.

**Method:**

this was a cross-sectional survey. A convenience sample of 326 currently practicing enrolled nurses (EN) or registered nurses (RN) was recruited. Respondents completed a questionnaire which was based on the implementation strategies advocated by the WHO Global Forum for Governmental Chief Nursing Officers and Midwives (GCNOMs). Questions covered the government initiative, healthcare financing policy, human resources policy, and the respondents' perception of importance and contribution of nurses in achieving UHC in elderly care services.

**Results:**

the knowledge of nurses about UHC in elderly care services was fairly satisfactory. Nurses in both clinical practice and management perceived themselves as having more contribution and importance than those in education. They were relatively indifferent to healthcare policy and politics.

**Conclusion:**

the survey uncovered a considerable knowledge gap in nurses' knowledge of UHC in elderly care services, and shed light on the need for nurses to be more attuned to healthcare policy. The educational curriculum for nurses should be strengthened to include studies in public policy and advocacy. Nurses can make a difference through their participation in the development and implementation of UHC in healthcare services.

## Introduction

Universal health coverage (UHC) is defined as the entire spectrum of health services, ranging from health promotion, disease prevention, acute care and treatment, rehabilitation, to palliative care, and it should be financially affordable and geographically accessible to everyone in need^(^ 1 ^)^. The definition embraces two key concepts: inclusiveness of the coverage and the sustainable development of the services provided.

Despite many political and resource constraints, the initiatives for UHC have been reinforced again in 2000, in many countries, since the establishment of Millennium Development Goals (MDGs) following the United Nations Millennium Summit^(^ 1 ^)^. One hundred and ninety-one United Nations members have committed to achieve the MDGs by 2015. In response to the MDGs, the WHO Global Forum for Governmental Chief Nursing Officers and Midwives (GCNOMs) has declared a commitment to develop a competent nursing workforce at all levels of healthcare delivery systems to support the initiatives for UHC^(^ 2 ^)^. A set of implementation strategies was subsequently recommended for countries to follow. To succeed, it requires the contribution of nurses who are involved in policy making, management, education and clinical service.

The WHO has been advocating UHC over the past few decades to ensure all human beings are able to seek health services and are not deprived of services because of financial hardship^(^ 3 ^)^. The elderly population is one of the most vulnerable groups that require extra effort in order to achieve UHC. This is partly because of the loss of gainful employment and partly because of the increased incidence of co-morbidity in this group of people. As expected, demands for health and social care will increase by many folds due to the trending rise in the aging population. Thus, the rights of elderly in accessing healthcare may face unprecedented levels of threat; Hong Kong is no exception^(^ 4 ^)^. In Hong Kong, the healthcare system, including elderly services, relies on both public sector and private sector. While 88% of the secondary and tertiary healthcare services were provided by the public sector, nearly 70% of the primary healthcare services were provided by the private sector^(^ 5 ^)^. All Hong Kong citizens are eligible to seek medical services from the public sector at a very low fee. This fee may also be waived if the person covered by the comprehensive social security scheme (CSSA).

The development of the nursing profession in Hong Kong is considered relatively more mature than in many Asian countries, yet the level of nurses' participation in politics was reported to be low^(^ 6 ^)^. More often than not, nurses were perceived to be apathetic to political decisions, even if they were healthcare related^(^ 7 ^)^. Heavy workloads, a sense of powerlessness, gender bias, lack of understanding of the political and policy making process, and ethical conflicts between professional and political values may account for this. Nurses, as one of the major healthcare providers, are the key members in the provision of quality healthcare services, and advocate for health choices and health policies^(^ 8 ^-^ 9 ^)^. It is important for them to be knowledgeable of the implementation strategies for UHC, even if they do not fully understand.

Healthcare services for elderly in Hong Kong are far from adequate, despite many new initiatives have been implemented^(^ 10 ^-^ 12 ^)^. Many institutions, such as day centers, skilled nursing facilities and infirmaries want to support the initiatives; however, they cannot find enough nurses to do so. The goal of achieving UHC for elderly healthcare services is moving farther away. The situation does not appear to have any impact on nurses. This is rather unusual, as nurses have been very devoted to vulnerable people in Hong Kong. Hence, the research team decided to look into the fundamental problem that leads to this phenomenon. Are nurses aware of these initiatives which were purposely developed to support UHC for elderly healthcare services in Hong Kong?

The UHC movement was first initiated in 1941. Over the last few decades, the focus of the movement has been reviewed and changed, for example, from poverty to gender equality, and to child welfare. However, the impact of UHC is yet to be seen. Engagement of various agencies, government officials, political leaders and relevant stakeholders is crucial in the course of implementation^(^ 13 ^)^. For engagement to succeed, knowledge of every party is crucial. To understand this, the research team designed this study to explore nurses' knowledge of Universal Health Coverage (UHC) for inclusive and sustainable development of elderly care services in Hong Kong.

## Method

A cross-sectional survey was conducted in May and June of 2015, after ethical clearance was approved by The Hong Kong Institute of Education. A list of potential respondents was generated from a pool of nurses who had experience interacting with some members of the research team. One researcher then called the respondents to explain the purpose of the call and the details of the study. He also checked their eligibility. Having obtained their consent to participate, the research team sent an information sheet and a questionnaire to the respondents electronically. Email reminders were sent to them two and four weeks after the initial distribution of the questionnaire. Names were not collected, to ensure anonymity.

To ensure ecological validity, the research team developed a demographic profile sheet and 17 questions initially based on the implementation strategies recommended by the WHO Global Forum for the Governmental Chief Nursing Officers and Midwives (GCNOMs). Three research team members who were not involved in the development of the questionnaire served as experts to independently review the relevancy of the draft questions. Four questions were removed and several required further elaboration by adding sub-questions to the original questions.

Apart from the demographic profile, there were two parts in the final version of the questionnaire, namely, knowledge of inclusiveness of UHC and the perceived contribution to sustainable development of UHC (Figure 1). Inclusiveness of UHC was composed of the government initiative (Q1), healthcare financing policy (Q2, 3, 4, 5 and 6), and human resources policy (Q7, 8, 9, 10 and 11). Respondents were asked to indicate their level of knowledge of UHC. For sustainable development of UHC, respondents were asked to rate their perceived contribution (Q12) and perceived importance of nurses (Q13). Split half reliability was performed using Spearman's coefficient which was satisfactory at 0.881. With the unique function of the e-questionnaire system, the respondents' answers were automatically compiled in a table format. Descriptive and inferential statistics were then computed and a comparison was performed by years of experience, job title, nature of one's role, and their qualifications.

### Survey on nurses' knowledge of universal health coverage (UHC) for inclusive and sustainable elderly care services in Hong Kong

**Figure 1:**
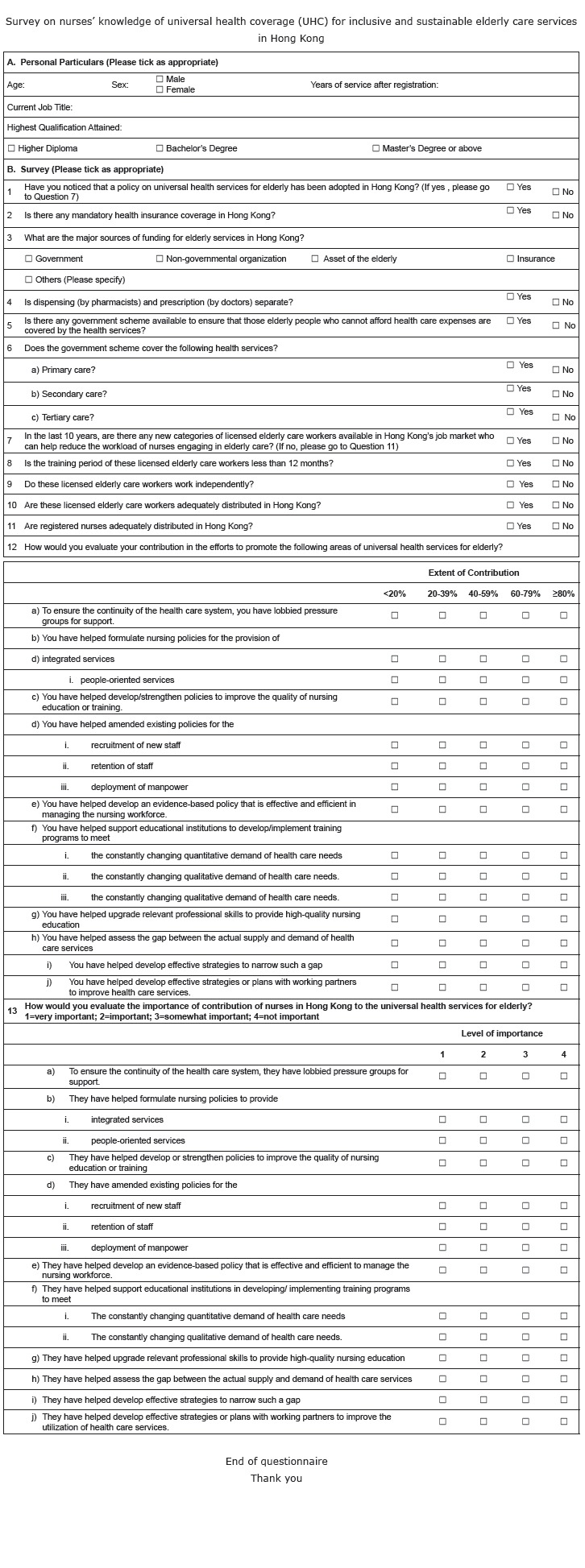
- Survey on nurses' knowledge of universal health coverage (UHC) for inclusive and sustainable elderly care services in Hong Kong

## Results

Of the 326 recruited respondents, about 75.8% were female (n=247) and 24.4% were male (n=79). The sample size provided reasonable protection against Type II error, given a minimum 5% of precision with 5.41% of alpha error, whereas the confidence interval was 95%.

### Demographic characteristics

Table 1 shows the demographic characteristics of the respondents. The ratio between registered nurse and enrolled nurse (RN/EN) was comparable to that of the proportional distribution in Hong Kong^(^ 14 ^)^. The majority of them were young (aged 35 or below), baccalaureate prepared, and working in clinical settings.

**Table 1:** - Demographic distribution of respondents (N=326). Hong Kong, 2015

**Variables**	**n**	**%**	
Gender (N=326)			
	Female	247	75.8
	Male	79	24.2
Age Group (N=320)			
	≦ 20	3	0.9
	21 - 25	91	28.4
	26 - 30	76	23.8
	31 - 35	76	23.8
	36 - 40	22	6.9
	≧ 41	52	16.3
Post-registration Year (N=326)			
	1 - 5	171	52.5
	6 - 10	76	23.3
	≧ 11	79	24.2
Current Job Title (N=326)			
	Enrolled Nurse (EN)	73	22.4
	Registered Nurse (RN)	192	58.9
	Senior Clinician (RN)	16	4.9
	Management (RN)	26	8.0
	Education (RN)	19	5.8
Nature of Role (N=326)			
	Clinician	269	82.5
	Management	39	12.0
	Education	18	5.5
Highest Qualification (N=326)			
	Higher Diploma	88	27.0
	Bachelor's Degree	136	41.7
	Master's Degree or Higher	102	31.3

*Percentage may not add up to 100 because some respondents declined to answer the questions.

### Inclusiveness of UHC for elderly healthcare services: Governmental initiative 

The results showed that slightly more than half of the respondents (171, 52.5%) were unaware of the implementation of UHC in Hong Kong. No statistical significances were reported by years of experience, job title, nature of role, or qualification.

### Inclusiveness of UHC for elderly healthcare services: Healthcare financing policy

Two hundred and eighty-eight (88.3%) respondents correctly answered that there was no mandatory health insurance coverage in Hong Kong. Significant statistical differences were detected between nurses with a Higher Diploma (HD) and nurses with a master's degree (p=0.005). More nurses with HD responded incorrectly that there was mandatory health insurance coverage in Hong Kong than nurses with a master's degree. These respondents (n=288) were asked to indicate their knowledge of the healthcare financing issues in questions 2 to 6 (Table 2) which covered the inclusiveness of UHC for elderly healthcare services.

**Table 2:** - Percentage of responses in healthcare financing. Hong Kong, 2015

**Question**	**By years of experience (%)**		**By job title (%)**	**By nature of role (%)**	**By qualification (%)**																	
	**1-5**	**6-10**	**>10**		**Sig.**		**EN**	**RN**		**Sig.**		**C**	**M**	**E**		**Sig.**		**H**	**B**	**M**		**Sig.**
2	86.5	90.8	89.9		NS		80.8	90.6		NS		87.7	94.9	83.3		NS		80.7	89.0	94.1		0.015
3	83.8	84.1	80.3		NS		84.7	82.8		NS		83.5	81.1	80.0		NS		80.3	89.3	77.1		NS
4	23.0	36.2	63.4		<0.001		25.4	30.5		<0.001		29.2	70.3	60.0		<0.001		19.7	28.9	57.3		<0.001
5	56.1	60.9	64.3		NS		49.2	58.6		NS		56.4	72.2	73.3		NS		53.5	62.8	58.9		NS
6a	7.2	11.9	19.6		NS		10.3	9.8		NS		9.8	14.8	27.3		NS		10.5	5.3	21.1		0.019
6b	72.3	66.7	84.8		NS		65.5	72.5		NS		71.4	85.2	81.8		NS		68.4	71.1	82.5		NS
6c	48.2	54.8	73.9		0.018		51.7	50.0		0.050		51.1	74.1	81.8		0.020		50.0	48.7	71.9		0.018

*Percentage only includes affirmative responses †NS = Not significant ‡By nature of role: C = Clinician, M = Management and E = Education §By qualification: H = Higher Diploma, B = Bachelor's degree, and M = Master's degree or higher ǁ Only those who answered 'No' to 2 were required to answer 3, 4, 5, 6a, 6b and 6c

About two-thirds of the participants indicated that dispensing and prescribing were separate systems (Q4), which was wrong. Forty percent of respondents incorrectly indicated that the government did not cover elderly who could not afford health services (Q5) and tertiary care (Q6c). In Hong Kong, the Government was not the major provider for primary care (Q6a), however, 88.3% thought it was.

### Inclusiveness of UHC for elderly healthcare services: Human resource policy

Enrolled Nurses (ENs) showed significantly higher level of knowledge of the presence of the new categories of licensed elderly care workers in Hong Kong than the RNs, while those with a HD had significantly better knowledge of the training period (Table 3). This is reasonable because ENs were mostly HD holders while RNs held degrees. It may also reflect the reality that many ENs work in the elderly care sector, where many care providers belong to the new category of licensed elderly care workers.

**Table 3:** - Percentage of responses in human resources policy. Hong Kong, 2015

**Question**	**By years of experience (%)**		**By job title (%)**	**By nature of role(%)**	**By qualification (%)**																	
	**1-5**	**6-10**	**>10**		**Sig.**		**EN**	**RN**		**Sig.**		**C**	**M**	**E**		**Sig.**		**H**	**B**	**M**		**Sig.**
7	57.3	50.0	50.6		NS		63.0	51.6		0.047		54.6	48.7	55.6		NS		56.8	55.9	49.0		NS
8	86.7	78.9	89.7		NS		91.3	82.8		NS		85.7	84.2	88.9		NS		96.0	82.9	79.6		0.043
9	36.7	34.2	41.0		NS		43.5	35.4		NS		38.1	36.8	22.2		NS		42.0	34.2	36.7		NS
10	74.5	73.7	84.6		NS		67.4	78.8		NS		74.8	88.9	84.2		NS		70.0	73.7	87.8		NS
11	19.9	19.7	14.1		NS		17.8	19.8		NS		19.0	17.9	11.8		NS		15.9	20.6	17.8		NS

*Percentage only includes affirmative responses †NS = Not significant ‡By nature of role: C = Clinician, M = Management and E = Education §By qualification: H = Higher Diploma, B = Bachelor's degree, and M = Master's degree or higher ǁOnly those who answered 'Yes' to 7 were required to answer 8, 9 and 10

### Sustainable development of UHC for elderly healthcare services: Perceived contribution 

Question 12 asked the respondents to evaluate the extent of their contribution in the efforts to promote UHC for elderly healthcare services (Figure 1). For the purpose of data analysis, the research team considered the respondent's rating of 40% or higher as 'having positive contribution'. Table 4 showed that those with six to ten years of experience, RNs and those with a master's degree or higher perceived themselves to have significantly less contribution in formulating nursing policies for the provision of integrated care, people-orientated care, amendments to existing policies for recruitment of new staff, and supporting educational institutions to develop/implement training programs to meet the societal needs qualitatively.

**Table 4:** - Percentage of responses in perceived contribution to UHC for elderly healthcare services. Hong Kong, 2015

**Question**	**By years of experience (%)**		**By job title (%)**		**By nature of role (%)**		**By qualification (%)**															
	**1-5**	**6-10**	**>10**		**Sig.**		**EN**	**RN**		**Sig.**		**C**	**M**	**E**		**Sig.**		**H**	**B**	**M**		**Sig.**
12a	30.4	15.7	29.1		0.025		43.8	19.3		<0.001		26.1	30.8	27.8		NS		32.9	24.2	24.5		NS
12b-i	43.9	23.6	35.5		0.001		56.1	29.8		0.001		36.8	43.6	27.8		NS		46.6	36.8	29.4		0.015
12b-ii	61.4	44.8	41.9		0.009		63.0	51.0		NS		53.9	46.1	50.1		NS		59.1	55.1	44.1		0.038
12c	44.4	36.9	48.1		NS		49.3	37.5		0.027		41.3	51.3	61.1		NS		42.0	41.8	43.5		NS
12d-i	33.9	18.4	34.1		0.026		42.5	25.5		0.029		30.5	35.9	16.7		NS		34.1	33.1	23.6		0.043
12d-ii	22.8	21.0	35.5		0.040		28.8	20.8		0.043		23.4	41.0	22.2		NS		21.6	28.0	25.4		NS
12d-iii	27.4	22.4	34.1		0.045		35.6	22.4		0.024		26.8	38.5	22.3		NS		23.8	33.1	24.5		NS
12e	29.3	18.4	36.7		NS		38.3	22.9		0.020		27.6	38.5	22.3		NS		29.6	28.6	27.4		NS
12f-i	44.4	32.9	53.1		0.007		52.0	35.9		0.002		40.8	53.8	66.7		0.031		45.5	41.2	46.1		NS
12f-ii	45.6	31.5	57.0		0.006		52.1	36.5		<0.001		41.2	59.0	72.2		0.005		44.3	44.1	47.1		NS
12g	59.1	47.4	63.3		NS		63.0	52.1		0.023		55.4	64.1	72.2		NS		61.4	53.8	58.8		NS
12h-i	40.3	34.2	37.9		NS		46.6	33.9		NS		37.8	46.2	27.8		NS		44.3	36.0	36.3		NS
12h-ii	31.6	25.0	27.8		NS		41.1	23.4		0.005		29.0	35.9	16.7		NS		37.5	25.7	26.5		0.049
12i	32.8	27.6	44.3		NS		42.4	27.0		0.017		32.0	53.8	27.8		NS		36.4	32.3	35.2		NS

*Percentage only includes extent of contribution >=40% †NS = Not significant ‡By role nature: C = Clinician, M = Management and E = Education §By qualification: H = Higher Diploma, B = Bachelor's degree and M = Master's degree or higher

### Sustainable development of UHC for elderly healthcare services: Nurses' importance 

Table 5 showed nurses' perceived importance to the sustainable development of UHC for elderly healthcare services. In general, those with 6-10 years of experience, RNs, in management and with a master's degree or higher showed significantly higher perceived contribution. Those in education showed the lowest perceived contribution in all aspects.

**Table 5:** - Percentage of nurses' perceived importance to sustainable UHC for elderly healthcare services. Hong Kong, 2015

**Question**	**By years of experience (%)**		**By job title (%)**	**By nature of role (%)**	**By qualification (%)**																	
	**1-5**	**6-10**	**>10**		**Sig.**		**EN**	**RN**		**Sig.**		**C**	**M**	**E**		**Sig.**		**H**	**B**	**M**		**Sig.**
13a.	79.5	84.2	86.1		NS		71.2	85.4		0.022		81.8	94.9	61.1		0.027		73.8	88.2	81.4		NS
13b-i.	84.2	93.5	91.1		0.009		80.9	89.6		NS		87.3	94.9	83.3		NS		85.3	86.8	92.2		NS
13b-ii.	86.6	97.4	92.4		NS		86.3	92.2		NS		90.7	92.3	83.3		NS		87.5	89.7	94.1		NS
13c.	85.4	98.7	93.7		<0.001		82.2	93.2		NS		90.4	94.8	83.4		NS		85.2	90.4	95.1		0.018
13d-i.	79.5	90.8	89.8		0.045		76.7	87.0		NS		84.4	92.3	72.2		0.032		80.7	83.1	90.2		NS
13d-ii.	83.0	93.4	93.6		NS		78.1	91.2		0.028		87.7	94.9	77.7		0.005		81.8	87.5	94.1		NS
13d-iii.	81.3	93.4	91.2		NS		79.4	88.0		NS		85.9	92.3	83.3		NS		83.0	83.1	94.1		NS
13e.	81.3	92.1	88.6		NS		78.1	88.5		0.035		85.9	89.7	72.2		0.023		80.7	86.1	89.2		0.027
13f-i.	83.0	90.8	88.6		NS		82.2	88.1		NS		86.6	87.1	77.7		NS		84.1	86.0	88.3		NS
13f-ii.	85.4	96.0	89.9		0.030		82.2	92.2		NS		89.6	89.7	77.7		NS		85.2	89.0	92.2		NS
13g.	86.6	96.0	92.4		0.029		82.1	94.3		NS		91.1	89.7	77.8		NS		87.5	91.1	91.2		NS
13h-i.	78.4	90.7	84.8		0.013		78.1	84.9		NS		83.3	87.2	66.7		NS		76.1	83.9	87.2		NS
13h-ii.	83.0	90.8	83.5		NS		80.8	88.0		0.027		86.2	82.0	72.2		0.031		84.1	85.3	85.3		NS
13i.	83.7	88.2	88.6		NS		79.4	89.6		0.005		87.0	87.2	66.7		0.012		80.7	89.0	86.3		NS

*Percentage only includes important and very important †NS = Not significant ‡By nature of role: C = Clinician, M = Management and E = Education §By qualification: H = Higher Diploma, B = Bachelor's degree, and M = Master's degree or higher

## Discussion

The respondents' knowledge of UHC for elderly healthcare services was fairly satisfactory. Those who were in clinical and management positions perceived themselves as having more contribution and importance in UHC implementation in comparison with the responses by the educators. Nurses were relatively indifferent to healthcare policy and politics. Possible explanations and implications will be discussed below.

### Inclusiveness of UHC for elderly healthcare services

In general, significantly more respondents incorrectly identified that we had an independent and separate drug-dispensing system in Hong Kong. They were those who had less years of experience, were ENs, in clinical practice, and held a diploma qualification. Similar findings were found in healthcare financing for tertiary care. Conversely, for healthcare financing in primary care, those with bachelor's degrees showed significantly lower level of knowledge of its source of funding. A separated drug-dispensing system has been debated in Hong Kong for over two decades, and its advocates have encountered enormous resistance from the medical profession. The low level of knowledge of this among nurses implies their remoteness from public affairs and policy in general. On the other hand, ENs were more aware of the existence of licensed elderly care workers than the RNs. Universal health coverage for elderly healthcare services has significant implications to our healthcare system. As our population ages, one in every three citizens in 2041 will be elderly^(^ 15 ^)^. The demand for healthcare services will increase. To meet the escalating demand, the government needs to allocate additional resources, be they human or financial, to prepare the society. The RNs constitutes the major nursing workforce in Hong Kong. There is no reason for RNs to have such low level knowledge and to be unprepared for this forthcoming challenge.

### Sustainable development of UHC for elderly healthcare services

This study revealed that nurses showed low political involvement and powerlessness in the process of policy making, which was consistent with the findings from previous studies^(^ 7 ^,^ 16 ^-^ 18 ^)^. Focusing on the difference between RNs and ENs, 88.5% of the former believed that it was important for nurses to develop evidence-based policy for managing the nursing workforce, while only 78.1% of the latter agreed that it was the nurse's role. This may reflect the importance of training and education in fostering nurses' political sense, particularly their understanding of the policy making process. From the core competencies stipulated by The Nursing Council of Hong Kong, ENs are only required to practice in accordance with policies,^(^ 19 ^)^while RNs are expected to understand the process of developing health care policies. However, only 15 hours were suggested for teaching health care policies in the curriculum for nursing education^(^ 20 ^)^. The inadequacy of policy studies in nursing education can be reflected in the answers of the respondents to the questions about the perceived contribution to evidence-based policy making. Only 38.3% and 22.9% of ENs and RNs, respectively, claimed that they had more than a 40% contribution in the development of evidence-based policy. This finding suggests that the majority of nurses feel powerless and remote from policy-making related to healthcare services. As a result, they are indifferent to the political process leading to UHC for elderly healthcare services in Hong Kong. This phenomenon warrants the immediate attention of the nursing profession. It may be timely and appropriate to reconsider the long standing suggestion to incorporate political education in the education of nurses^(^ 7 ^,^ 16 ^-^ 18 ^)^.

From the findings of this study, nurses with higher academic qualifications, such as the master's degree and higher, perceived a significantly higher level of importance in helping to develop/strengthen policies to improve the quality of nursing education. In recent years, the Government has proposed several major changes in elderly care policy in response to the challenges evolving from our rapidly aging society, such as strengthening primary care, emphasizing aging in place, and a voluntary health insurance scheme. Understandably, these changes mean increasing demand for both RNs and ENs at the community level. The question is: will nurses be able to meet the demand, or an even better question may be, have nurses been prepared for it? Unfortunately, with the present RN and EN mix, the answer is negative. The findings of this study reaffirmed this. It is crucial, therefore, to involve nurses in policy-making, particularly when a major change is expected to occur. To ensure nurses are competent in the political process, the professional body such as The Nursing Council of Hong Kong should consider revising the indicated nursing curriculum and core-competency of ENs and RNs to strengthen the nurses' knowledge and ability to participate in policy development. Thus, the gap between policy and practice could be bridged.

Despite the leading role of nursing education in Asia, surprisingly, nurse educators perceived relatively low importance of nurses' contribution to healthcare services for the elderly in Hong Kong, as compared to their counterparts in clinical practice and management. It may imply that nurse educators felt that they had minimal power in affecting the healthcare system and policy-making in Hong Kong. Another possible explanation may be that this is due to the low involvement of nurse educators in setting policy and, needless to say, the political agenda^(^ 21 ^)^ Nurse educators may only have a chance to voice their view if there is an interest group or a public consultation. Since nurse educators are responsible for nurturing the future generation of nurses, they should be role models for their students, and should equip themselves well in this area. There is an urgent need to involve more nurse educators in the political process leading to decision-making^(^ 22 ^)^.

The survey uncovered a considerable knowledge gap in nurses' knowledge of UHC in elderly care services, but care must be taken in interpreting the findings from such a nonrandom sample. Having collected the data on nurses' perceived contribution and importance to policy-making across clinical, management and education sectors, the research team believes that, with the increase of the aged population, nurses could do more to enhance their capacity at various fronts to support the government's initiatives to provide UHC for elderly healthcare services^(^ 23 ^)^.

## Conclusion

Universal health care evolves from the 'Health for All' movement advocated by the WHO in the 1970s. Since then, the Hong Kong government has launched many initiatives in order to achieve UHC, particularly for inclusive and sustainable elderly healthcare services. Although the outcomes of these initiatives are yet to be seen, the research team considered it to be appropriate to conduct the reported survey to identify nurses' knowledge of and involvement in the process, including policy-making and implementation. It was hoped that the findings would inform major stakeholders of some issues which may possibly affect the success of these initiatives.

The survey has revealed some knowledge gaps among nurses. Their knowledge of healthcare financing, including health insurance, drug-dispensing, and human resource policy needs to be enhanced. The low perceived importance and contribution to the sustainable development of elderly healthcare services are deterrents to their possible involvement in the initiatives. After all, nurses constitute a major work force in healthcare. They should be better prepared to participate with policy-making knowledge for the benefit of the population that they serve.
